# Do the Number, Size, and Position of Partially Threaded Screws Affect the Radiological Healing of Surgically Treated Displaced Femoral Neck Fractures? A Review of 136 Children

**DOI:** 10.3390/medicina58091153

**Published:** 2022-08-25

**Authors:** Wentao Wang, Zhu Xiong, Chongzhi Zhao, Bo He, Haibo Mei, Yiqiang Li, Federico Canavese, Yuancheng Pan, Shunyou Chen

**Affiliations:** 1 Scientific Research Center, The Seventh Affiliated Hospital, Sun Yat-sen University, Shenzhen 518107, China; 2 Department of Pediatric Orthopedics, Shenzhen Children’s Hospital, Shenzhen 518034, China; 3 Department of Pediatric Orthopedics, Foshan Hospital of Traditional Chinese Medicine, Foshan 528099, China; 4 Department of Orthopedics, Children’s Hospital of Chongqing Medical University, National Clinical Research Center for Child Health and Disorders, Ministry of Education Key Laboratory of Child Development and Disorders, Chongqing 400015, China; 5 Department of Pediatric Orthopedics, Hunan Children’s Hospital, Changsha 410007, China; 6 Department of Pediatric Orthopedics, Guangzhou Women and Children’s Medical Center, Guangzhou Medical University, Guangzhou 510623, China; 7 Lille University Center, Jeanne de Flandre Hospital, Department of Pediatric Orthopaedics, Avenue Eugène Avinée, 59037 Lille, France; 8 Department of Pediatric Orthopedics, Fuzhou Second Hospital, 47th Shangteng Road of Cangshan District, Fuzhou 350007, China

**Keywords:** femoral neck fracture, children, fracture healing, radiographs, cannulated screws, quantity, size, position

## Abstract

*Background and Objectives:* The quantity, size, and position of implants might affect the fracture healing process of surgically treated displaced pediatric femoral neck fractures (PFNFs). The aim of this retrospective multicenter study was to evaluate the correlation between the time needed to achieve radiological union and the number, size, and location of the partially threaded cannulated screws (PTCSs) in children with displaced PFNFs. *Materials and Methods:* A retrospective review of 136 children (mean age: 10.6 ± 3.8 years) with displaced PFNFs treated by two (*n* = 103) or three (*n* = 33) PTCSs was carried out. Student’s *t*-tests, one-way ANOVA, Cox regression analysis, and multiple linear regression analyses were performed to investigate the variables affecting the time needed to achieve radiological fracture healing according to the number, size, and position of PTCSs, as assessed on plain radiographs. *Results:* A total of 132 hips achieved union at an average of 3.2 ± 1.6 months after the initial surgery. The time needed to achieve union in the patients treated with two or three PTCSs was comparable (*p* = 0.36). Among the fractures treated by two PTCSs, the time needed to achieve union did not correlate with the size of the implant (*p* = 0.122), or with the angulation between the PTCSs on anterior–posterior (*p* = 0.257) and lateral radiographs (*p* = 0.547). The time needed to achieve union in the fractures that were fully compressed by the implants was similar to the partially compressed fractures (*p* = 0.08). *Conclusions:* The number, size, and position of the PTCSs do not affect the radiological healing in the children with displaced PFNFs treated surgically.

## 1. Introduction

Pediatric femoral neck fractures (PFNFs) are relatively rare, although the rate of complications, such as delayed union, nonunion, and avascular necrosis (AVN) of the femoral head, is not negligible [1,2,3]. Internal fixation by partially threaded cannulated screws (PTCSs) is recommended in children with PFNFs requiring surgical treatment [4,5].

Studies on adult patients with displaced femoral neck fractures have reported that the biomechanical stability tends to increase with the quantity and size of the implants, and have indicated that a higher number of larger cannulated screws should be used in the surgical treatment of such injuries [6,7,8]. However, several other reports came to opposite conclusions and reported that a lower number of smaller screws would significantly decrease the risk of AVN in patients with displaced PFNFs undergoing surgical treatment [9,10]. This controversy may be attributed to the fact that the previous studies did not evaluate the correlation between the time needed to achieve fracture healing and the quantity, size, and position of the implants in the surgically treated PFNFs [11]. In addition, it was shown that when all of the screw threads were within the proximal fragment of the fracture, the screws provided full fracture compression and decreased the incidence of nonunion in the adults with femoral neck fractures that were treated surgically [12,13]. However, having all of the threads above the fracture line may not be possible in the children with displaced PFNFs due to the proximity of the growth plate to the fracture and the risk of iatrogenic growth arrest if the implants cross the growth plate. In addition, the softer trabecular bone that is typically seen in children can further limit the compression exerted by the screw at the fracture site [14]. To our knowledge, none of the previous reports have evaluated the correlation between the time needed to achieve fracture union and the quantity, size, and position of PTCSs in the children with displaced PFNFs treated surgically.

The aim of this retrospective multicenter study was to evaluate the correlation between the time needed to achieve radiological union and the quantity, size, and direction of PTCSs in the children with displaced PFNFs treated surgically.

## 2. Materials and Methods

After securing Institution Review Board approval, we retrospectively reviewed the medical records of 545 children with displaced PFNFs that were treated surgically from March 2010 to June 2020 at 4 institutions in China.

The inclusion criteria were as follows: (1) diagnosis of unilateral displaced PFNF and the absence of other concomitant injury; (2) a type II or type III PFNF according to the Delbet–Colonna classification [15]; (3) surgical treatment by closed or open reduction and internal fixation with 2 or 3 PTCSs; (4) age at surgery <17 years; (5) patients without any additional injuries or nutritional metabolic diseases; (6) a follow-up >6 months; (7) complete clinical data; and (8) complete radiographic data, including standard postoperative anterior–posterior (AP) and cross-table or frog-leg lateral pelvic radiographs and consecutive postoperative radiographs with a follow-up interval <2 months [16,17].

**Figure 1:**
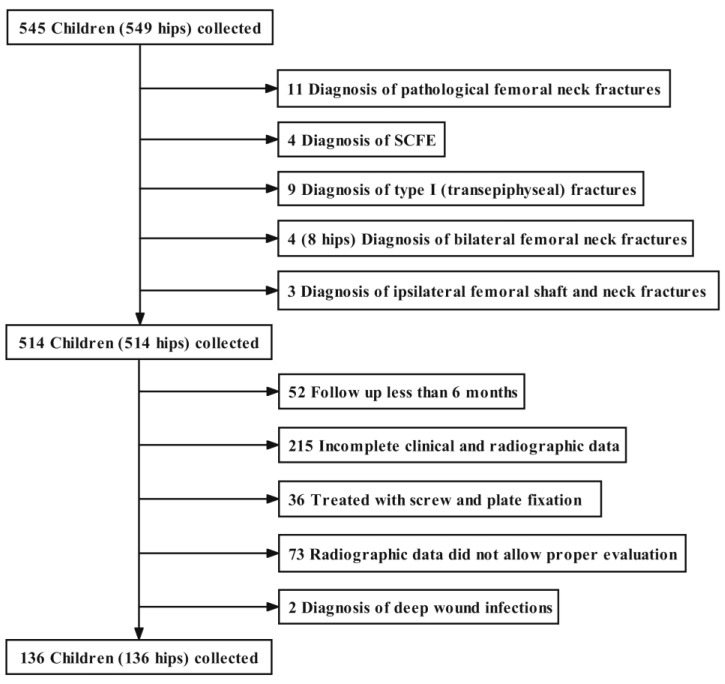
Study flowchart.

A total of 136 out of 545 children (25%) met the inclusion criteria; there were 79 (58.1%) boys and 57 (41.9%) girls. The remaining 409 patients (75%) were excluded for the following reasons: diagnosis of a pathological femoral neck fracture (*n* = 11; 2%); a slipped capital femoral epiphysis (SCFE) (*n* = 4; 0.7%); a Delbet–Colonna type I fracture (*n* = 9; 1.7%) [15]; bilateral fractures (*n* = 4; 0.7%); ipsilateral femoral shaft and neck fractures (*n* = 3; 0.6%); a follow-up <6 months (*n* = 52; 9.5%); incomplete clinical and radiographic data (*n* = 215; 39.4%); fracture treated with screw and plate fixation (*n* = 36; 6.6%); radiographs of poor quality not allowing proper evaluation (*n* = 73; 13.4%); and a diagnosis of deep wound infection (*n* = 2; 0.4%) (Figure 1).

The mean age at injury was 10.6 ± 3.8 years (range, 2–17); 77 fractures involved the left side (56.6%) and 59 (43.4%) involved the right side. All of the injured hips were preoperatively immobilized in a brace.

According to the system described by Delbet and adapted by Colonna [15], 86/136 patients (63.2%) had Delbet–Colonna type II (trans-cervical) fractures, and 50/136 (36.8%) had Delbet–Colonna type III (cervico-trochanteric) fractures. The severity of the initial displacement was evaluated according to the system reported by Wang et al. [18]. In particular, 22 PFNFs (16.2%) were classified as type I (incomplete fractures with any translation or an angulation <30°), 77 (56.6%) as type II (complete fractures with any angulation and translation <50%), and the remaining 37 (27.2%) as type III (complete fractures with any angulation and translation >50%).

Eighty-two hips (60.3%) underwent closed reduction (CR) and fixation by 2 (*n* = 57; 41.9%) or 3 (*n* = 25; 18.4%) PTCSs, and the remaining 54 (39.7%) underwent open reduction (OR) and fixation by 2 (*n* = 46; 33.8%) or 3 (*n* = 8; 5.9%) screws. Overall, 103 fractures (75.7%) were treated with 2 PTCSs and 33 (24.3%) were treated with three PTCSs. In addition, none of the patients undergoing CR had a joint decompression, while those managed by OR underwent a joint decompression by opening the hip joint along the anterior part of the femoral neck.

According to Song et al.’s classification system [5], the quality of reduction was rated as follows: (1) anatomical: PFNF without any residual interfragmentary displacement or angulation; (2) acceptable: PFNF with a residual interfragmentary displacement <2 mm or an angular deformity <20°; (3) unacceptable: PFNF with a residual interfragmentary displacement >2 mm or an angular deformity >20°. Overall, 65 PFNFs (47.8%) were anatomical, 66 (48.5%) were acceptable, and 5 (3.7%) were unacceptable reductions.

Following the operation, all of the hips were immobilized within a spica cast or brace with the affected hip in a valgus position. The patients were reviewed every one month before the fracture achieved radiological healing. At each follow-up visit, the radiographs were used to evaluate the radiological healing. The fractures were defined as healed (radiological union) if the callus, trabeculae, and bone bridged the fracture site on the four cortices on the AP and lateral radiographs [3,19,20,21]. The spica cast or brace was removed until the fracture had achieved radiological union.

**Figure 2:**
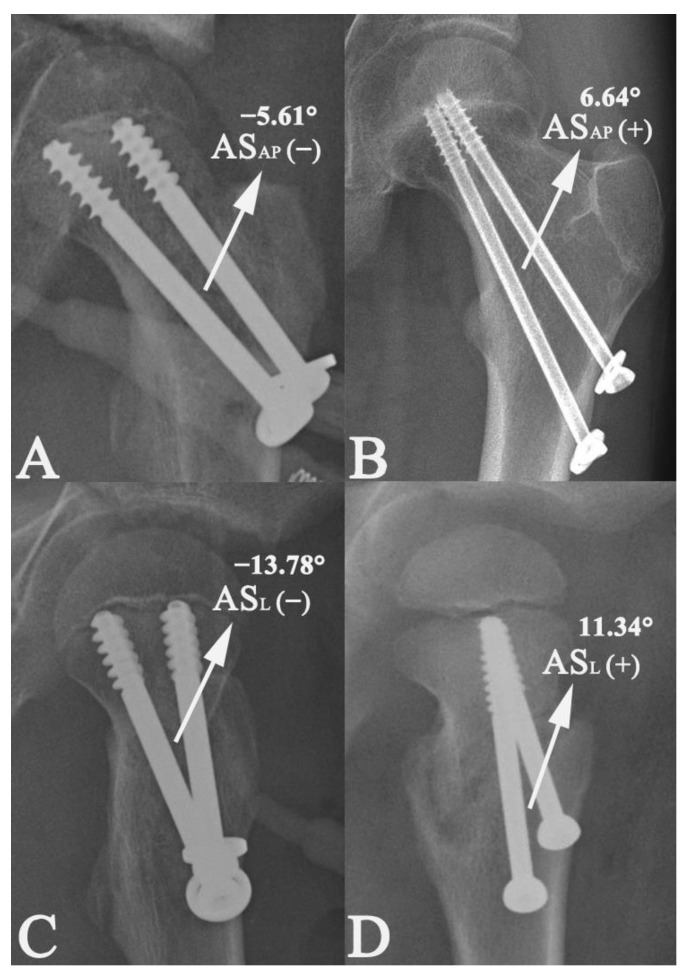
The angulation (AS) between the axis of two cannulated screws on anterior–posterior (AS_AP_) and lateral (AS_L_) radiographs were both measured and were expressed as degrees (°). The value of AS_AP_ or AS_L_ was defined as neutral (zero) if two screws were parallel, negative if two screws were divergent (**˅**-shape), and positive if two screws were convergent (**˄**-shape) (**A**–**D**).

The period of time between the surgical treatment and the follow-up visit at which the radiological union of the fracture was first defined was recorded as the time needed to achieve radiological union, and was expressed in months. The ratio of the PTCS diameter to the femoral neck width was calculated; in particular, the PTCS diameters made it possible to relate this measurement to the width of the femoral neck, thus making it possible to calculate the ratio, which was then expressed as a percentage (%). The angulation (AS) between the axis of the two cannulated screws on the AP (AS_AP_) and lateral (AS_L_) radiographs were both measured and were expressed as degrees (°). The value of AS_AP_ or AS_L_ was defined as neutral (zero) if the two screws were parallel, negative if the two screws were divergent (**˅**-shape), and positive if the two screws were convergent (**˄**-shape) (Figure 2).

**Figure 3:**
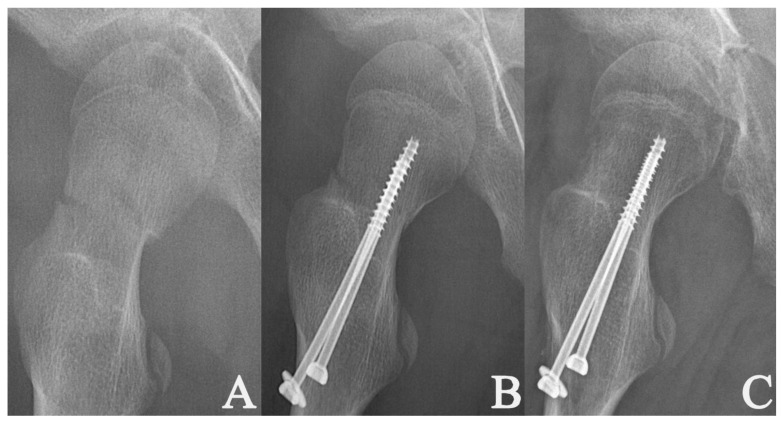
Radiographs of a 9-year-old girl with a displaced Delbet–Colonna type II fracture [15] (**A**); radiographs after closed reduction and internal fixation with two cannulated screws filling 11.9% of the femoral neck; the fracture was partially compressed by the hardware (**B**); the radiological healing at 1.9 months post-surgery (**C**).

Whether the fracture fragments were fully or partially compressed by the PTCSs was evaluated according to the location of the screw threads in relation to the fracture site. The fracture fragments were considered fully compressed if all of the threads were above the fracture line (within the proximal fragment) on the AP and lateral postoperative radiographs (Figure 2). If one or more of the threads were at the level of the fracture line or below the fracture line on either AP or lateral radiographs, the fracture fragments were defined as partially compressed (Figure 3).

All of the radiographic measurements were performed using the picture archiving and communication systems (PACS; GE Healthcare, Chicago, IL, USA) by two experienced pediatric orthopedic surgeons (WWT and LYQ), and the mean values were used for the statistical analysis.

### Statistical Analysis

The statistical analysis was performed using the statistical package SPSS 13.0 (SPSS, Chicago, IL, USA).

The data are expressed as numerical variables, frequencies, and percentages, and as the means and standard deviations. The Student’s *t*-tests were used to evaluate the correlation between the time needed to achieve fracture healing and sex, laterality, type of fracture, medial or posterior comminuted cortex on the AP or lateral radiographs, the method of reduction, the quality of reduction, number of PTCSs implanted, and whether the fracture fragments were fully or partially compressed. One-way ANOVA was performed to assess the correlation between the time needed to obtain fracture union and the severity of the initial displacement, mechanism of injury, and quality of reduction. A Cox regression analysis was used to assess the factors influencing the probability of, and the time needed to, achieve a radiological fracture union. When performing the Cox regression analysis, the final event was defined as achieving radiological fracture union, and the variable ’time’ was the time needed to achieve radiological union. On the other hand, the patients not achieving radiological fracture healing at the final follow up were recorded as censoring, and for them, the variable ’time’ corresponded to the follow up time. Multiple linear regression analysis was used to evaluate the effect of several numerical variables, including age, time to reduction, implant size, and implant position, on the time needed to achieve fracture union. The level of statistical significance was set at *p* < 0.05.

## 3. Results

**Figure 4:**
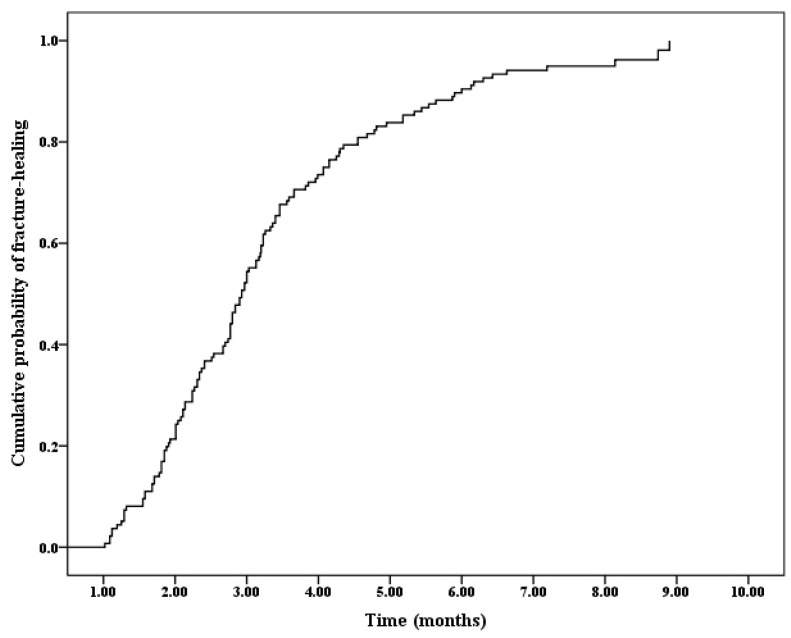
Overall probability of achieving fracture healing according to the Cox regression analysis.

A total of 132 (97.1%) out of 136 hips had consolidated (radiological healing) during the follow-up (Table 1). Four of the hips (2.9%) were recorded as censored data, due to the failure to achieve fracture union at the final visit with a follow-up duration of 7.6 ± 0.7 months (range, 6.7–8.2). Cox regression analysis results indicated that the overall probability of achieving fracture healing increased with the follow up time (Figure 4). The mean time required to obtain fracture union and the duration of the spica cast or brace application was 3.2 ± 1.6 months (range, 1–8.9). The time needed to achieve radiological fracture healing according to the subgroup analysis is shown in Table 1.

### 3.1. Time Needed to Achieve Healing in the Fractures Treated by Two or Three PTCSs (n = 132)

The average age of the patients at the time of injury was 10.6 ± 3.8 years old (range, 2–17). The average time from injury to surgical treatment was 4.7 ± 3.6 days (range 1–19). Multiple regression analysis showed no significant difference between the time to achieve fracture healing and both age (*p* = 0.069) and time to surgery (*p* = 1.000).

**Figure 5:**
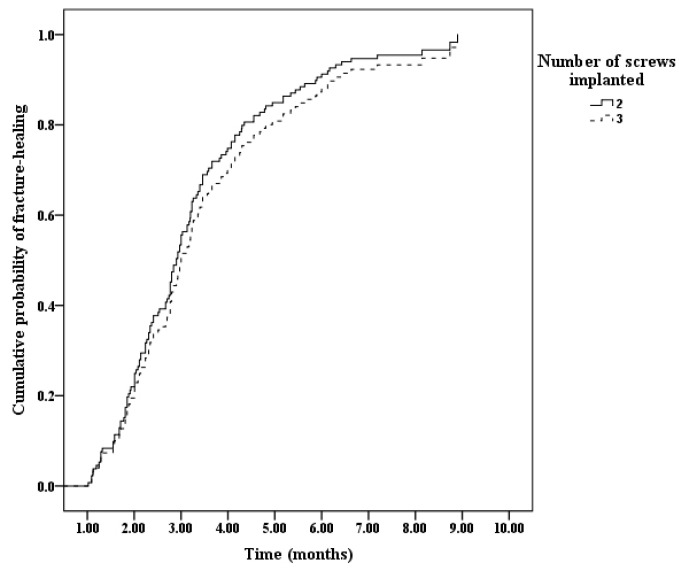
Cumulative probability of achieving radiological fracture healing according to the number of screws implanted.

A severe initial displacement (*p* = 0.008), comminuted medial or posterior cortex on the AP or lateral radiographs (*p* = 0.035), and poor quality of reduction (*p* < 0.001) were significantly associated with a longer time needed to achieve fracture healing; other factors, such as sex (*p* = 0.749), laterality (*p* = 0.538), mechanism of injury (*p* = 0.243), type of fracture (*p* = 0.243), method of reduction (*p* = 0.901), and number (two versus three) of PTCSs (*p* = 0.36), were not associated with a longer time to achieve radiological union (Table 1). Cox regression analysis also confirmed these results (Table 2 and Figure 5). The subgroup analysis indicated that the time to achieve fracture healing in the PFNFs treated with two PTCSs was comparable to that of the PFNFs treated with three PTCSs, regardless of the severity of the initial displacement, the comminution of the medial or posterior cortex, and the quality of the reduction (Table 3).

### 3.2. Time Needed to Achieve Healing in the Fractures Treated by Two PTCSs (n = 100)

The mean implant/width of the femoral neck percentage was 18 ± 3.1 (range 11.9–28.8). The average AS_AP_ and AS_L_ were 1.3° ± 4.3° (rang, −12.5° to 16.7°) and −2.2° ± 5.1° (range −21° to 12.4°), respectively. Multiple linear regression analysis indicated no significant correlation between the time needed to achieve union and the size of the implant (*p* = 0.122), AS_AP_ (*p* = 0.257), or AS_L_ (*p* = 0.547) (Table 4).

Seventy-two fractures (72%) were fully compressed, and twenty-eight (28%) were partially compressed by two PTCSs. The Student’s *t*-tests indicated no significant difference regarding the time needed to achieve fracture healing between the fully and partially compressed PFNFs (*p* = 0.08) (Table 1).

## 4. Discussion

The current study investigated the correlation between the time needed to achieve fracture union and the number, size, and position of implants in children with displaced PFNFs that were repaired by two or three PTCSs. We found that the number of screws implanted did not affect the radiological time needed to achieve radiological fracture healing. Additionally, the size of the implant, the direction (parallel, convergent, or divergent) of the screws, and whether the fracture was fully or partially compressed were not variables affecting the time needed to achieve radiological union in the patients treated by two PTCSs.

**Figure 6:**
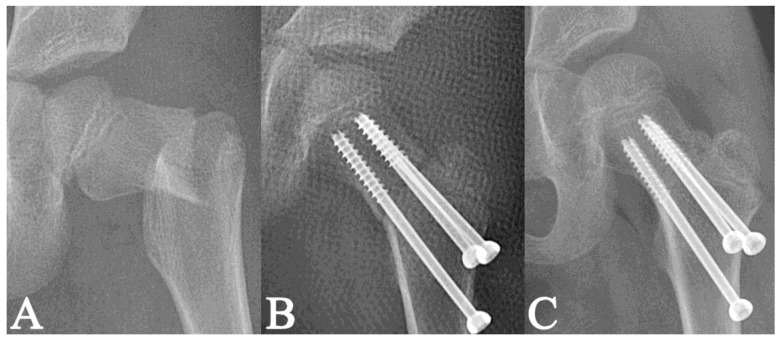
Radiographs of a 5-year-old girl with a displaced Delbet–Colonna type II fracture [15] (**A**); radiographs after closed reduction and internal fixation with 3 cannulated screws (**B**); the radiological healing at 3.9 months post-surgery (**C**).

Our study showed that the time needed to achieve fracture healing in the children with PFNFs treated with two PTCSs was comparable to that of those treated with three PTCSs (Figure 6). This finding means that two screws can achieve sufficient mechanical stability to maintain the reduction and promote fracture healing. However, Eberl et al. reported opposite conclusions [22]. In their study of 22 displaced PFNFs treated by internal fixation with an average of 2.4 cannulated screws, a loss of reduction occurred in 12 (54.5%) patients, and they concluded that the screw fixation could not provide sufficient mechanical stability [22]. This problem may be related to the lack of a spica cast or brace immobilization post-surgery in their study [22]. In our study, all of the patients underwent a spica cast or brace immobilization following surgery, and only one case of secondary displacement (0.7%) was observed. Our findings are in accordance with previous studies that reported excellent outcomes in patients with displaced PFNFs treated by two screws and cast or brace immobilization [23,24]. In addition, the use of three PTCSs may not be recommended in the pediatric population due to the increased risk of damaging the intra-osseous vascular supply of the proximal femur during implant insertion. Wang et al. reported that the fractures managed by three screws are associated with a higher incidence of AVN of the femoral head than those treated by two screws [9].

Interestingly, our study also found that the size of the implant did not correlate with the time needed to achieve radiological fracture healing. This finding may be attributed to the larger amount of cancellous bone in the pediatric patients. Other works have shown that the cancellous bone present in the femoral neck of children, due to its greater density than that of adults, provides a sufficient grip for the threaded screws, or even just threaded K-wires [25,26]. Therefore, this finding may explain why fewer and smaller PTCSs can provide sufficient mechanical stability to achieve fracture healing without increasing the risk of AVN [9].

**Figure 7:**
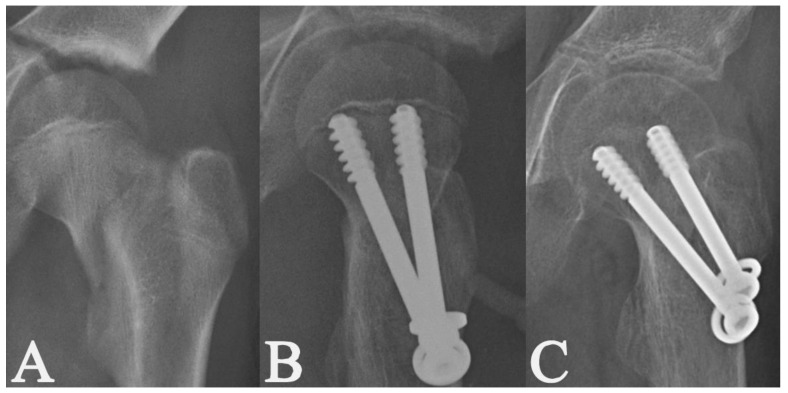
Radiographs of a 12-year-old boy with a displaced Delbet–Colonna type III fracture [15] (**A**); radiographs after open reduction and internal fixation with 2 cannulated **˅**-shape screws; the fracture was completely compressed by the screws with the threads above the fracture line (**B**); the radiological healing at 3.5 months post-surgery (**C**).

**Figure 8:**
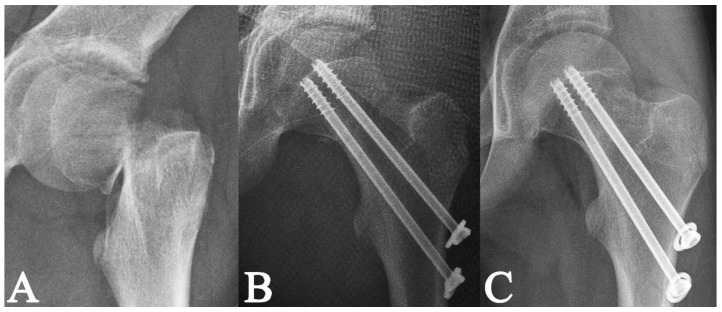
Radiographs of a 12-year-old boy with a displaced Delbet–Colonna type II fracture [15] (**A**); radiographs after open reduction and internal fixation with 2 cannulated **˄**-shape screws; the fracture was completely compressed by the screws with the threads above the fracture line (**B**); the radiological healing at 2.8 months post-surgery (**C**).

The current study indicated that the screw position (parallel, divergent, or convergent) did not affect the time needed to achieve fracture healing (Figure 7 and Figure 8). This result is in agreement with the findings of Lim et al. [27]. They reviewed 445 patients with femoral neck fractures that were fixed with parallel (*n* = 195) or nonparallel (*n* = 250) cannulated screws, and no difference was found regarding the rate of nonunion, irrespective of the screw direction [27]. Spangler et al. also reported similar results [28]. On the other hand, Uhl and Le Corroller et al. reported different findings, although none of these works were based on clinical data [29,30]. In our opinion, cannulated screws with parallel or nonparallel directions can both provide sufficient mechanical stability. As a result, the surgeon, at the time of screw insertion, does not have to spend too much time aiming for an optimal, parallel implant direction. This reduces the operative time and the number of perforations into the cancellous bone of the femoral neck, which can damage the intraosseous vascular system supplying the femoral head [9].

Our study did not show any difference in the time needed to achieve fracture union according to the amount of compression (full or partial) exerted by the PTCSs. This finding is in contrast with previous reports that focused on femoral neck fractures in adult patients [12,13]. These studies indicated that fully compressed fractures heal faster than partially compressed fractures due to the increased mechanical stability [12,13]. This controversy could be attributed to the different trabecular patterns within the femoral neck as well as the different cortical thicknesses between children and adults. Several studies have shown that children younger than 12 to 13 years of age do not have the typical and rigid adult trabecular pattern, which can strengthen the compression force exerted by cannulated screws [14,31]. In our study, most of the patients were younger than 11 years of age (mean age: 10.6 years), and a rigid trabecular pattern was not present in these patients. In addition, having all of the threads above the fracture line may increase the risk of violating the growth plate, especially for the patients with fracture lines that are closer to the growth plate.

It should be noted that we encountered limitations in the analysis of our results. First, this was a retrospective study. Second, we failed to identify the smallest implant diameter that should be used in children with displaced PFNFs without compromising the fracture stability. Third, biomechanical evaluations were not performed. Despite these limitations, this is the first study that evaluated the clinical association between the time needed to achieve fracture healing and the number, size, and position of implants in children with displaced PFNFs treated by two or three PTCSs.

## 5. Conclusions

In conclusion, the displaced PFNFs treated by two PTCSs have a comparable time needed to achieve radiological fracture healing to that of the displaced PFNFs treated by three PTCSs. The size of the implant, direction (parallel, convergent, or divergent) of the PTCSs, and whether the fracture is fully or partially compressed by the screws do not affect the time needed to achieve radiological fracture healing in the fractures treated by two screws.

## Figures and Tables

**Table 1:** Time needed to achieve fracture healing according to the demographics of the patients.

		Time (Months)	t/F	*p*
Gender	Male (*n* = 78; 59.1%)	3.2 ± 1.7	0.321	0.749
	Female (*n* = 54; 40.9%)	3.3 ± 1.5		
Laterality	Left (*n* = 75; 56.8%)	3.3 ± 1.7	0.618	0.538
	Right (*n* = 57; 43.2%)	3.1 ± 1.4		
Initial displacement according to Wang et al. [18]	Type-I (*n* = 22; 16.7%)	2.3 ± 1.2	4.960	0.008 *
	Type-II (*n* = 74; 56.1%)	3.3 ± 1.5		
	Type-III (*n* = 36; 27.3%)	3.5 ± 1.9		
Mechanism of injury	Ta (*n* = 52; 39.4%)	3.2 ± 1.5	1.410	0.243
	Fa (*n* = 33; 25.0%)	2.8 ± 1.2		
	Sp (39; 29.5%)	3.6 ± 2.0		
	Ots (8; 6.1%)	3.4 ± 1.7		
Type of fracture [15]	Delbet–Colonna I (*n* = 0; 0%)	-	1.174	0.243
	Delbet–Colonna II (83; 62.9%)	3.1 ± 1.8		
	Delbet–Colonna III (*n* = 49; 37.1%)	3.4 ± 1.3		
	Delbet–Colonna IV (*n* = 0; 0%)	-		
Medial or posterior cortex on AP or lateral radiographs	Comminuted (*n* = 24; 18.2%)	3.8 ± 1.8	2.133	0.035 *
	Without comminution (*n* = 108; 81.8%)	3.1 ± 1.5		
Reduction method	CRIF (*n* = 79; 59.8%)	3.2 ± 1.6	0.125	0.901
	ORIF (*n* = 53; 40.2%)	3.2 ± 1.7		
Fixation method	Two cannulated screws (*n* = 100; 75.8%)	3.1 ± 1.6	0.919	0.360
	Three cannulated screws (*n* = 32; 24.2%)	3.4 ± 1.5		
Quality of reduction according to Song et al. [5]	Anatomical (*n* = 65; 49.2%)	2.6 ± 1.2	12.098	<0.001 *
	Acceptable (*n* = 63; 47.7%)	3.7 ± 1.7		
	Unacceptable (*n* = 4; 3.0%)	5.3 ± 2.4		
Fractures fixed by two cannulated screws	Fragments fully compressed (*n* = 72; 72%)	3.0 ± 1.6	1.771	0.08
	Fragments partially compressed (*n* = 28; 28%)	3.6 ± 1.6		

AVN: avascular necrosis; AP: anterior–posterior; Ta: traffic accident; Fa: fall; Sp: sport; Ots: others; CRIF: closed reduction and internal fixation; ORIF: open reduction and internal fixation; An: anatomical; Ac: acceptable; Uac: unacceptable. *: *p* < 0.05.

**Table 2:** Risk factors for achieving fracture union by Cox regression analysis.

	Coefficient	SE	Wald	*p*	RR	95% of CI
Initial displacement	−0.288	0.146	3.910	0.048 *	0.750	0.564, 0.997
Comminuted medial or posterior cortex on AP or lateral radiographs	−0.500	0.234	4.558	0.033 *	0.607	0.383, 0.960
Quality of reduction	−0.708	0.168	17.769	<0.001 *	0.493	0.355, 0.685

AP: anterior–posterior; SE: standard error; RR: relative risk; *: *p* < 0.05; CI: confidence interval.

**Table 3:** Time needed to achieve fracture healing in children treated by 2 or 3 screws according to the initial displacement, comminution of the medial or posterior cortex, and quality of reduction.

		Number of Screws Implanted	Time Needed to Achieve Fracture Healing (Months)	t	*p*
Initial displacement	Type I	2	2.3 ± 1.2	0.871	0.394
		3	1.6 ± 0.4		
	Type II	2	3.2 ± 1.5	1.11	0.271
		3	3.6 ± 1.6		
	Type III	2	3.6 ± 2.0	0.258	0.798
		3	3.4 ± 1.2		
Medial or posterior cortex on AP or lateral radiographs	Comminuted	2	3.9 ± 2.0	0.28	0.782
		3	3.7 ± 1.4		
	Without comminution	2	3.0 ± 1.5	0.928	0.356
		3	3.3 ± 1.6		
Quality of reduction	Anatomical	2	2.6 ± 1.2	0.534	0.596
		3	2.8 ± 1.3		
	Non-anatomical	2	3.8 ± 1.8	0.139	0.89
		3	3.8 ± 1.6		

AP: anterior–posterior; PFNFs: pediatric femoral neck fractures.

**Table 4:** Multiple linear regression analysis of the time needed to achieve fracture union with the screw diameter and position of implants in patients treated with 2 screws.

	Coefficient	SE	t	*p*	95% CI
Screw’s diameter (%)	0.081	0.052	1.561	0.122	−0.022, 0.184
AS_AP_ (°)	−0.047	0.041	−1.140	0.257	−0.129, 0.035
AS_L_ (°)	−0.021	0.035	−0.605	0.547	−0.090, 0.048

AS_AP_: the angle between two cannulated screws on AP views; AS_L_: the angle between two cannulated screws on lateral views; SE: standard error; CI: confidence interval.
